# Association of Gestational Age at Birth with Reasons for Subsequent Hospitalisation: 18 Years of Follow-Up in a Western Australian Population Study

**DOI:** 10.1371/journal.pone.0130535

**Published:** 2015-06-26

**Authors:** Ravisha Srinivasjois, Claudia Slimings, Kristjana Einarsdóttir, David Burgner, Helen Leonard

**Affiliations:** 1 Department of Paediatrics, Joondalup Health Campus, The University of Western Australia, Perth, Western Australia, 6027, Australia; 2 Telethon Kids Institute, The University of Western Australia, Perth, Western Australia, 6008, Australia; 3 Murdoch Childrens Research Institute, Flemington Road, Parkville, Victoria, 3052, Australia; 4 Department of Paediatrics, University of Melbourne, Parkville, Victoria, 3052, Australia; 5 Department of Paediatrics, Monash University, Clayton, Victoria, 3168, Australia; Vanderbilt University, UNITED STATES

## Abstract

**Background:**

Preterm infants are at a higher risk of hospitalisation following discharge from the hospital after birth. The reasons for rehospitalisation and the association with gestational age are not well understood.

**Methods:**

This was a retrospective birth cohort study of all live, singleton infants born in Western Australia between 1^st^ January 1980 and 31^st^ December 2010, followed to 18 years of age. Risks of rehospitalisation following birth discharge by principal diagnoses were compared for gestational age categories (<32, 32–33, 34–36, 37–38 weeks) and term births (39–41weeks). Causes of hospitalisations at various gestational age categories were identified using ICD-based discharge diagnostic codes.

**Results:**

Risk of rehospitalisation was inversely correlated with gestational age. Growth-related concerns were the main causes for rehospitalisation in the neonatal period (<1 month of age) for all gestational ages. Infection was the most common reason for hospitalisation from 29 days to 1 year of age, and up to 5 years of age. Injury-related hospitalisations increased in prevalence from 5 years to 18 years of age. Risk of rehospitalisation was higher for all preterm infants for most causes.

**Conclusions:**

The highest risks of rehospitalisation were for infection related causes for most GA categories. Compared with full term born infants, those born at shorter GA remain vulnerable to subsequent hospitalisation for a variety of causes up until 18 years of age.

## Introduction

Preterm birth is usually defined as gestational age (GA) at birth of less than 37 completed weeks and is recognised as an important risk factor for infant morbidity and mortality.[1] The immature brain, lungs, gastrointestinal tract as well as complications experienced in the neonatal period make these infants susceptible to long-term morbidities, including neurodevelopmental problems.[2,3] The risks are highest for infants born very preterm (GA<32 weeks) and the risks of short-term morbidity,[4] long-term morbidity [2] and mortality [5] decrease with increasing GA at birth.

Caring for premature infants consumes considerable resources. In the US, preterm infants account for around half of hospitalisation costs within the first year of life and a third of the hospitalisation costs until 18 years of age.[6] Hospitalisations within the first year of life are highest for very preterm infants [7–11] but decrease with increasing GA.[12] However, infants born moderately preterm, between 32 to 35 completed weeks of gestational age, remain at high risk of infectious and developmental morbidities compared with those born at term,[13–15] and, as such, the public health impact of this group is high and under-appreciated.[14]

There is limited information regarding the most common causes for rehospitalisation following discharge from the hospital after birth. In the neonatal period, respiratory disease is the most common reason for hospitalisation for very preterm infants,[16,17] whereas jaundice and feeding problems are the most common in infants born after 33 weeks.[13,15] Beyond the neonatal period, respiratory admissions predominate across all gestational ages up to 5 years of age.[7,9–12,18–21] However, there is generally a paucity of information regarding the reasons for rehospitalisation in childhood and adolescence, and its association with gestational age at birth. We have previously reported that the increased risk associated of hospitalisation in children born preterm persists into adolescence,[22] but the reasons for admission have not previously been investigated. Therefore we aimed to investigate the reasons for hospitalisations until 18 years of age according to gestational age categories at birth.

## Methods

This was a retrospective birth cohort of all live, singleton births in Western Australia (WA) between 1^st^ January 1980 and 31^st^ December 2010. Children were followed from the birth admission discharge to age 18 years, 31^st^ December 2010 if aged <18 years, or until death.

With 2.52 million residents, WA represents 11% of the Australian population.[23] Birth records from the Midwives Notification System were used to identify all children born in WA between 1^st^ January 1980 and 31^st^ December 2010. The records were linked to the Hospital Morbidity Data System (HMDS) and the Mortality Register. The HMDS collects information on all hospital admissions in WA, including the recording of diagnoses using the International Classification of Diseases (ICD) coding system. Since routinely collected deidentified data were used for the analysis, no individual written or informed consent was considered necessary. Ethical approval was provided by the WA Department of Health Human Research Ethics Committee (*2011/64*).

All hospital admissions occurring after discharge from the birth-related admission until 18 years were considered, including those originating in the first month of life but excluding those associated with normal birthing episodes (ICD-9-CM codes V30-V39). Hospitalisation episodes requiring admission to a special care nursery during birth hospitalisation were also excluded. Hospital admissions that were serial transfers (the patient moved between hospitals successively without returning home), nested transfers (the patient moves to another hospital during a stay in hospital), or an overlapping transfer (admission date prior to discharge date on previous record) were considered a single event. However, no information as to whether the admission was elective or emergency was available. The Australian modification of the 10^th^ revision (ICD-10-AM) was introduced in 1999; prior to this date the Australian version of the International Classification of Diseases, 9^th^ revision, Clinical Modification (ICD-9-CM) was used by hospitals to code diagnoses. ICD-10-AM codes were converted to best match ICD-9-CM codes using a backwards mapping table and grouped into 13 diagnostic categories based on previous work (S1 Appendix).[24] Only the principal diagnosis was used for analysis as it likely represents the primary reason for admission.

Gestational age was categorised according to current consensus guidelines.[13,25] Very preterm was defined as ≤32 completed weeks, moderate preterm as 32–33 completed weeks, late preterm as 34–36 completed weeks, early term as 37–38 completed weeks, term as ≥39 weeks.

### Statistical analysis

The percentages of live-born singleton children who were hospitalised at least once for all admissions and for different disease categories were calculated. Relative risks and their 95% confidence intervals were calculated for each gestational age group using births ≥39 weeks as the reference category and adjusted for sex and year of birth using generalised linear regression with a log link and binomial distribution. Analyses were conducted for admissions at different stages of childhood: neonatal period (0–28 days of age), post-neonatal period (29 days-1 year), infancy (1–5 years), childhood (5–12 years) and adolescence (12–18 years) excluding those who had died prior to the start of each age interval. Age intervals were calculated according to the date they became that age, e.g. 29 days-1 year was 29 days of age up to the date they turned 1 year, and 1–5 years was the day after turning one year to the date they turned five.

## Results

There were 765,069 singleton live births in WA between 1980 and 2010. Six individuals were excluded due to possible linkage errors leaving 765,063 for analysis. Of these, 473,493 (61.9%) were hospitalised at least once by 18 years of age, excluding birth admissions; 16.2% in the first 28 days, 16.9% between 29 days to 1 year, 32.7% between 1–5 years, 28.8% between 5–12 years, and 28.4% between 12–18 years of age (S1 Table). Gestational age was inversely associated with risk of hospitalisation; more than 90% of those born at ≤33 weeks gestational age were rehospitalised in the first 18 years, compared to 59% of infants born at ≥39 weeks (S1 Table).

**Fig 1:**
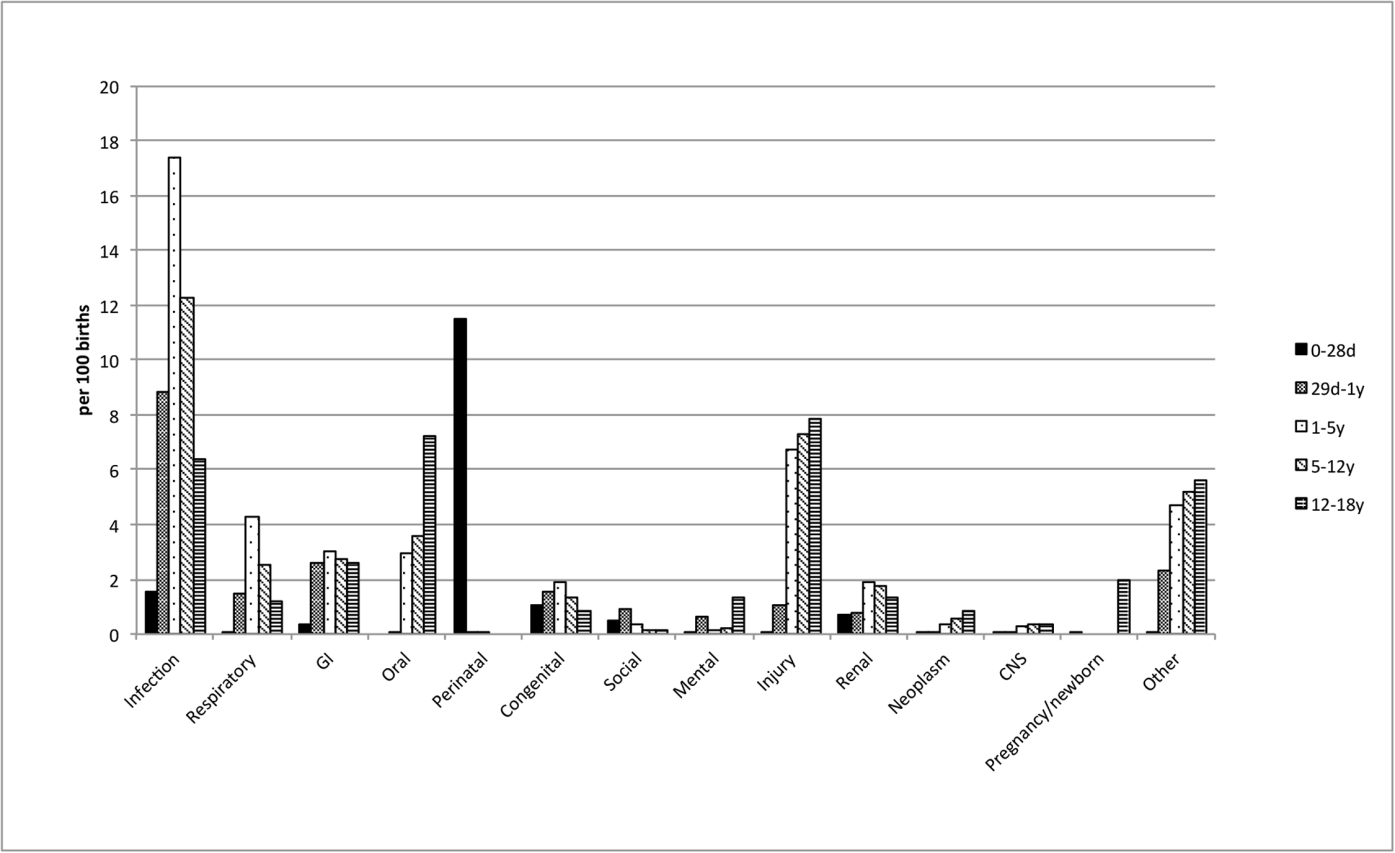
Admission rates (per 100 births) for each diagnosis category at each gestational age strata.

**Fig 2:**
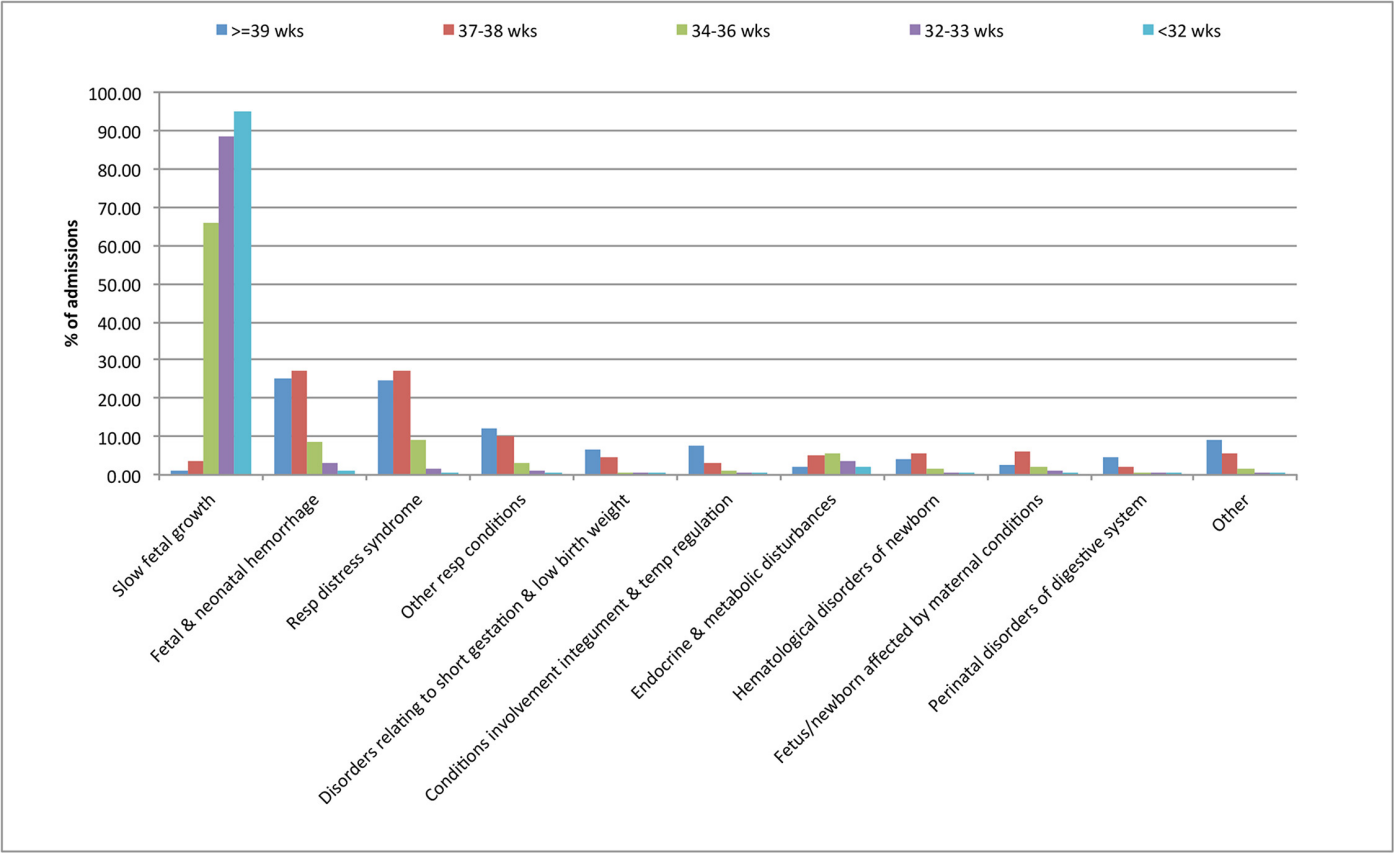
Most common diagnoses associated with perinatal admissions 0–28 days.

**Fig 3:**
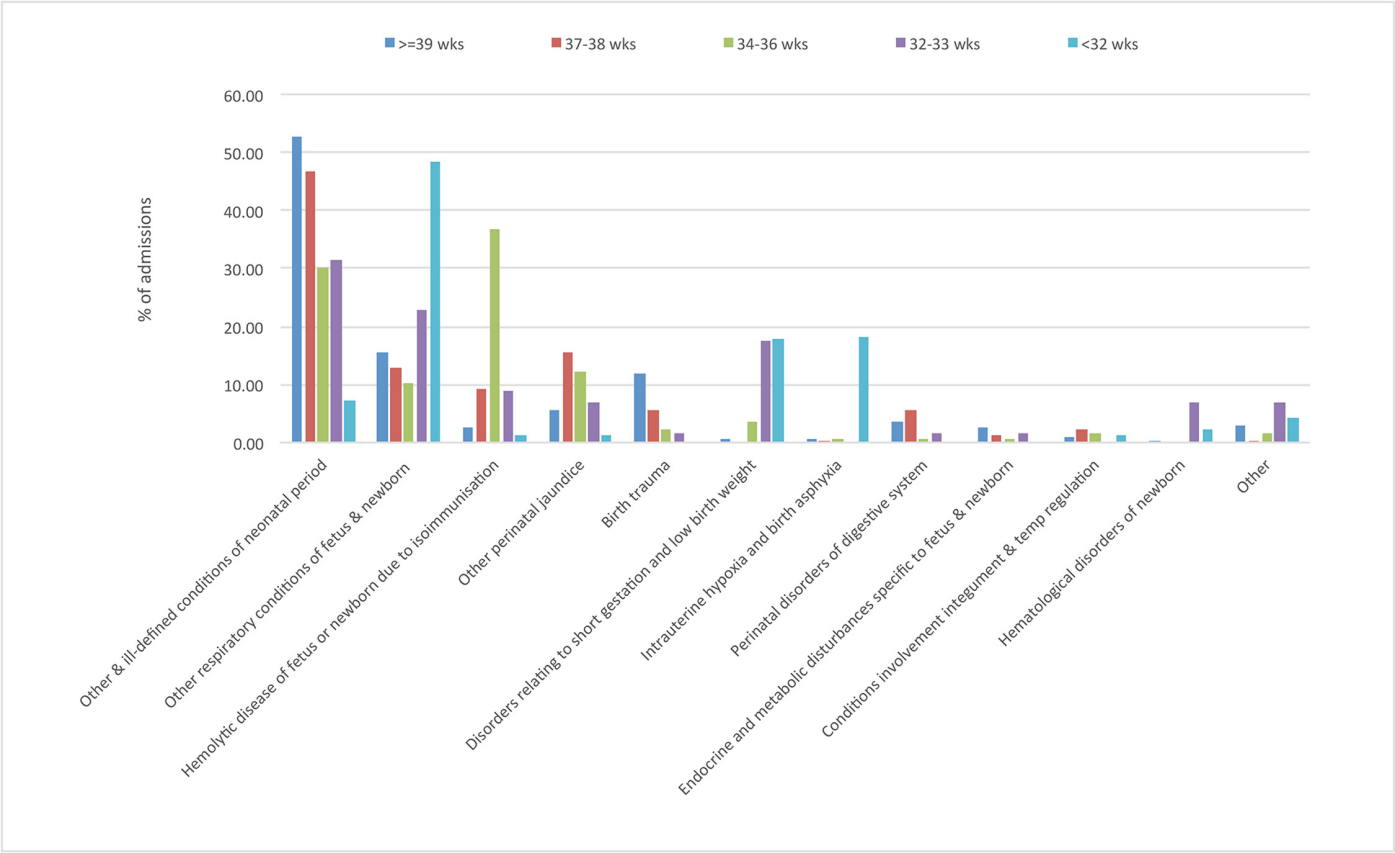
Most common diagnoses associated with perinatal admissions 29 days-1year.

Hospitalisation 0–28 days of life: The risk of rehospitalisation in the first 28 days of life was inversely related to gestational age (S2 Table, S2 Appendix). The risk was highest for infants born at <32 weeks compared with those born at >39 weeks. The predominant reason for rehospitalisation during the first 28 days was for conditions arising in the perinatal period, for which 11% of infants were hospitalised. For perinatal conditions a graded relationship between shorter gestational age and increased risk of hospital admission was observed (S2 Table). The most common perinatal condition was growth related (Fig 1) across all the GA categories with > 90% of infants born at less than 32 weeks of gestation requiring rehospitalisation on this account (Figs 2 and 3). In contrast to the pattern for perinatal conditions, the increased risk of rehospitalisation for infection, gastrointestinal and respiratory problems was seen for the early term and late preterm gestational ages (S2 Table, Figs 2 and 3).

Hospitalisation between 29 days-1 year: In this age group, infection was the most common reason for hospitalisation with 8.9% of children admitted at least once with infection, followed by gastrointestinal disorders (2.6%), and respiratory-related causes (1.5%) (S2 Appendix). For infants born at 37–38 weeks gestational age, the most common reason for hospitalisation was infections, followed by gastrointestinal and congenital-related causes, while for those born at <37 weeks, infectious, gastrointestinal and respiratory related conditions were common (S3 Table, S2 Appendix). Respiratory-related conditions were more common as the gestational age decreased (S3 Table, S2 Appendix). Infants born at shorter gestational ages were at increased risk of subsequent hospitalisation for the majority of diagnostic categories compared to those born at term. The largest relative risks were observed for perinatal, central nervous system, respiratory and gastrointestinal problems, and infection (S2 Appendix). The most common individual diagnoses for hospitalisation in each causal category are depicted in Figs 2 and 3 and S3 Appendix. For those aged between 29 days and one year, these included acute bronchitis and bronchiolitis in the infection category, asthma in the non-infectious respiratory category, nutrition-related causes and inguinal hernia in the gastrointestinal category and epilepsy and recurrent seizures in the central nervous system category (S3 Appendix).

Hospitalisation between 1–5 years: In this age group, 17.4% were hospitalised because of infection, followed by 6.7% for injury and 4.3% for a respiratory conditions (Fig 1). As in younger age groups, the risk of hospitalisation was inversely related to gestational age (S3 Table, S2 Appendix). For all gestational age strata, infection, injury and respiratory causes were the commonest causes for hospitalisation (S3 Table). A graded increase in the risk of central nervous system and injury related hospitalisation was observed as the gestational age decreased (S3 Table, S2 Appendix). There were marked associations between admissions for social causes in those between of 34–38 weeks gestational age, and for central nervous system causes in those <34 weeks gestational age. A graded relationship between shorter gestational age and the risk of infection-related admissions was also observed. The most common individual diagnoses included otitis media and infection of tonsils and adenoids in the infection category (S3 Appendix), asthma in the non-infection category, gastroenteritis and inguinal hernia in the GI category, and epilepsy and recurrent seizures in the central nervous system category (S3 Appendix).

Hospitalisation between 5–12 years: In this age range, infection remained the most common reason for hospitalisation, with 12.3% of the cohort admitted at least once with an infection, followed by injury (7.3%), and oral cavity-related diagnoses (3.6%) (Fig 1). The relationship between shorter gestational age and increased risk of hospitalisations for most diagnostic categories persisted. Common causes for hospitalisation included infection, injury, and respiratory related causes at all gestational ages (S3 Table). For infants born at <34 weeks of gestation the highest risks of hospitalisation compared with those born at term were from central nervous system-related causes, particularly epilepsy and recurrent seizures (S3 Appendix). Cerebral palsy-related diagnoses were also common in those born at <32 weeks gestation.

Hospitalisation between 12–18 years: In this age group, injury and oral cavity-related admissions were common, with 7.8% and 7.2% of children admitted respectively, while 6.3% of children were admitted on account of infection. The strongest associations with shorter gestational age were observed for central nervous system and social causes in those born at less than 36 weeks gestational age. Epilepsy and recurrent seizures, and migraine were the most common central nervous system causes for hospitalisation in this age group (S3 Appendix).

## Discussion

In this study, we explore the relationship between gestational age and birth and the risk of rehospitalisation up until the age of 18 years following discharge from the hospital after birth. We present data from over 765,000 live births over 20 years. Almost 62% of the cohort were rehospitalised at least once during childhood, with a striking inverse relationship with gestational age; over 90% of those born <33 weeks gestational age were readmitted to hospital. The reasons for admission, taken from the primary discharge diagnostic code, varied with both postnatal age and gestational age. Overall perinatal causes were the most common reason for hospitalisation in the first 28 days after birth discharge at all gestational ages. Infectious diseases were the most common cause of hospitalisation from 28 days onwards until 12 years of age, after which injury and oral cavity admissions became more common.

There is a considerable literature on the risk of hospitalisation post discharge from neonatal intensive care units.[13] Compared to full term infants this risk is higher even for late preterm (35–36 weeks) and early term (37–38) week infants. Using linked data on more than 6.6 million infants born in California, Ray et al. studied the risk of hospitalisation of late preterm and term infants during the first year of life.[26] They reported that the odds of any hospitalisation in the first 90 days of discharge post-birth hospitalisation decreased with increasing gestational age, after adjusting for maternal factors and for neonatal characteristics such as infant gender and growth status. Causes such as hyperbilirubinaemia and nonspecific perinatal causes were observed in late preterm infants compared with full term infants. Within the first 365 days post discharge, the risks of hospitalisation for respiratory, non-infectious respiratory, and gastrointestinal conditions decreased with increasing gestational age. In this study, respiratory infection was the most common cause for hospitalisation across all the gestational age categories in the first year after discharge.[26] Similar findings were reported studies of late preterm and early term infants in the US.[17] The risk of hospitalisation through the Emergency Department was highest in late preterm infants in the first 30 days after discharge and jaundice and feeding problems predominated. In the current study, perinatal causes and infections were the predominant causes of hospitalisation in the first 28 days of life. The risk was higher for shorter gestational ages. There was a persistent strong relationship with gestational age and admission for perinatal conditions even up to the age of one year. Further analysis of our data revealed the cause for most admissions in the first 28 days after hospital discharge was growth related. A recent report from the Canadian Neonatal network also indicated that small for gestational age infants experienced more neonatal complication and have a higher risk of hospital admissions compared to appropriately grown infants.[27]

Infection, mainly respiratory related is one of the commonest causes for hospitalisation in childhood. Preterm infants are at a higher risk of rehospitalisation due to sequelae of conditions such as chronic lung disease and necrotising enterocolitis.[27] Hospitalisation of French Canadian children under two years with lower respiratory infection was associated with a two fold increase in the risk of subsequent chronic respiratory morbidity up to the age of ten years.[28] In a Welsh cohort of 318,613 children born between 1998 and 2008, the risk of emergency hospitalisation for respiratory causes was inversely related to gestational age.[29] This risk was further increased in infants who were also small for gestational age.[29]

It has also been observed that low birth weight infants are at a higher risk of developing otitis media in view of reduced immunity and physiological immaturity of the Eustachian tube.[30,31] We observed that hospitalisation due to infection and conditions such as otitis media remained higher until early childhood across all gestational age strata compared with births ≥39 weeks. Bentdal et al. also found that the risk of acute otitis media in the first 18 months of age increased with decreasing GA when compared with infants born ≥37 weeks.[32] Similar to our results, the adjusted relative risk was higher for preterm infants compared with full term infants.[32]

Our analyses also identified injury as an important cause for hospitalisation across all gestational ages from 28 days onwards. Up until 12 years the risk of hospitalisation due to injury-related conditions was marginally higher for all gestational age strata compared with those born at term. A recent report from Sweden using nationwide data evaluated the risk of preterm infants experiencing unintentional injuries. In a study of 2,297,134 individuals, unintentional injuries were identified in 244,021 but in contrast to our findings, no relationship with preterm birth was observed.[33] In a snapshot of the health of Australian children between 2004 and 2005, respiratory disease (25%) and injury (12%) were the main causes of hospitalisation in children aged 1–14 years.[34] However relationships with gestational age were not reported.

One of the main strengths of our study include near complete linked statewide data and two decades of follow-up. More than two-thirds (78%) of the WA population live in the capital city, Perth, which is similar to the relative urban/regional distributions seen for Victoria (76% living in the state capital Melbourne) and South Australia (77% living in the state capital Adelaide), but greater than that seen for other Australian states and territories (range 48%-64%). The population in this study was identified from administrative data routinely collected on the WA population. De-identified data were linked and extracted by the Data Linkage Branch (DLB) of the WA Department of Health. The DLB links records between numerous datasets mainly held by the State government using probabilistic matching.[35,36] Evaluation of the data linkage system has shown that matching procedures are 99.89% accurate.[36] Outward migration in WA is estimated to be low (~3%) (personal communication, A/Professor Anna Ferrante). Therefore, we believe that our data captures the true trend in rehospitalisation with minimal data loss.

Our study does have a number of limitations. We relied on the primary discharge diagnosis coded and it is possible that this may not represent the main reason for hospitalisation. This issue could only be definitively resolved by retrieving a subset of the medical charts, not feasible in a study of this scope and nor permitted under the terms of our ethical approvals. The clinical practices and threshold for hospitalisations may also have changed over time. Information on risk factors such as socio-economic status, indigenous status and access to primary care services was not available, nor was it possible to distinguish between elective and emergency hospital admissions. There may also be overlap within the clinical coding, for example, respiratory causes and central nervous system causes may also include infections that may dilute the total number identified in each category. With respect to the analytical methods, we used generalised linear regression with a log link and binomial distribution to determine the relative risks for different causes of hospitalisation and adjusted for year of birth. However, considering that lengths of follow-up time varied among individuals within each hospitalisation age group, ignoring the time element in the analysis could have had some effect on the results even though the influence of birth year was removed.

Given the vast geographical area of Western Australia (approximately the size of India and Pakistan), access to preventative services such as outpatient clinics and community outreach clinics may be suboptimal, especially in rural areas. This may decrease the threshold for hospitalisation when such patients present to a regional centre, or mean that patients present late, when the disease is more severe. Separating out those living in the country from those in the metropolitan area was not possible due to the nature of the data. There also may be differences in health-seeking behaviour in families of infants and children born preterm (compared to term), that are impossible to quantify using population-level data. Our data included all live singletons but did not differentiate between healthy and chronically unwell preterm survivors for whom the risk of recurrent admission is likely increased.[37] A proportion of these infants could have had genetic or other conditions which could have predisposed them to prematurity and associated later comorbidity. Further studies should examine the role of these factors and additional characteristics of the birth admission, including growth status, on the risk of rehospitalisation in infancy and childhood.

Management of infants in the neonatal period, especially the very preterm infants, has changed significantly over the years. Introduction of surfactant in the 1990s, together with advances in respiratory support have resulted in increased survival of these very preterm infants.[38] Therefore the burden of chronic respiratory conditions, and central nervous system-related conditions may increase as overall survival improves.[39] However, at least in the first year of life, we have not found an increase over time in the risk of rehospitalisation for any gestational category, and relative to term births, the rehospitalistion rate has actually fallen other than for those born very pre-term.[22] Nevertheless, both direct and indirect costs associated with rehospitalisation is high and efforts should be made to minimise rehospitalisation by setting up support services such as community clinics aimed at preventative care.

## Conclusion

In conclusion there has been a lack of large population-level data of the impact of early gestation on subsequent morbidity as measured by the rate and types of hospitalisations throughout infancy, childhood and adolescence. Our study therefore aimed to provide such a comprehensive analysis. Our data are important to inform policy regarding preventative interventions and prioritisation of service provision. The findings have clear translational importance, especially if similar patterns are identified in other populations in whom analogous data are available. The striking finding is the high rates of rehospitalisation, even in those born late preterm/early term. Understanding the reasons for readmission at different ages is important in guiding the development of preventative interventions targeting those born preterm. These may include strategies that aim to optimising feeding and growth in infancy, prevent infections in the pre-school age group (e.g. by maintaining immunisation coverage), and prevent injuries in the school-age group. Successful interventions would reduce the hospitalisation rates in this population, with potentially considerable economic and health benefits.

## Supporting Information

S1 Appendix ICD 9 coding details. (DOCX)

S2 Appendix Risk of admission to hospital for different age categories. (DOCX)

S3 Appendix Common diagnosis for different age categories. (DOCX)

S1 Table Hospital admissions per 100 live, singleton births 1980–2010 over different childhood ages according to gestational age. (DOCX)

S2 Table Risk and causes for admission to hospital from birth to 28 days and 29 days-1 year by principal diagnosis and gestational age. (DOCX)

S3 Table Risk and causes for admission to hospital from 1–18 years by principal diagnosis and gestational age. (DOCX)
