# Supplementary material for: Association of Gestational Age at Birth with Reasons for Subsequent Hospitalisation: 18 Years of Follow-Up in a Western Australian Population Study
Source: PLoS One. 2015 Jun 26;10(6):e0130535. doi: 10.1371/journal.pone.0130535 (PMC4482718; doi:10.1371/journal.pone.0130535)
Supplement: S1 Appendix — (DOCX) [file pone.0130535.s001.docx]

Appendix 1.

ICD9 codes grouped into diagnostic categories

| Category | ICD-9 codes included in the category |
| --- | --- |
| Infection | 001-139; 320-326; 357.0; 372.0; 373.4-373.5; 376.0-376.1; 380.1; 381.0-381.5; 382; 383.0-383.3; 383.9; 390-398; 420-422; 460-466; 473-475; 480-490; 494; 510; 511.0-511.1; 523.3; 540; 567.0-567.2; 580; 590; 595; 598.0; 599.0; 601; 603.1; 604; 607.2; 608.0; 608.4; 614; 616.0-616.4; 646.6; 658.4; 675; 680-686; 711; 728.0; 730; 771.3-771.8; 780.3; 780.6; 790.7; 795.3; 795.7; V09; (exclude 090) |
| Non-infectious respiratory | 470-472; 476-478; 491-493; 495-508; 511.8-519; 782.5; 786; 793.1; 794.2; V12.6; V42.6; V44.0; V55.0; V71.2; (exclude 516.9; 786.6) |
| Gastrointestinal tract | 456.0-456.2; 530-578; 579.1-579.4; 782.4; 783; 787; 789; 790.4-790.5; 792.1; 793.3-793.4; 793.6; 794.8; V12.7; V18.5; V42.7; V44.1-V44.4; V45.3; V47.3; V53.5; V55.1-V55.4 (exclude 540; 567.0-567.2) |
| Oral cavity | 520-529; V41.6; V52.3; V53.4; V58.5; V72.2 (exclude 523.3, 524.0) |
| Perinatal period | 760-779, V13.7 (excludes 762.8; 771.0-771.8; 776.5; 778.0; 778.6) |
| Congenital anomalies | 090; 228.0-228.1; 243; 255.2; 270.0-271.3; 272.6-272.7; 275.1-275.4; 276.3; 277.0-277.2; 277.5; 282; 286; 288.2; 330; 335.0; 359.0-359.3; 362.7; 371.5; 389.1; 457.8; 524.0; 579.0; 579.8-579.9; 593.0; 593.7; 648.5; 654.0; 654.6-654.8; 740-759; 762.8; 771.0-771.2; 776.5; 778.0; 778.6; V13.6; V19.5; V82.4 |
| Social admissions | V20; V60-V65 (excluding V62.8-V62.9) |
| Mental health | 290-319; V11; V17.0; V18.4; V40; V62.8-V62.9; V66.3; V67.3; V71.0; V79 |
| Injury and poisoning | 800-999; V15.5-V15.6; V54.0-V54.2; V66.4; V67.4; V71.3-V71.6; V82.5 |
| Renal/genital | 250.4; 581-629; 646.2; 788; 791; 793.5; 793.8; 794.4; V13.0; V18.6-V18.7; V43.5; V44.5-V44.6; V45.1; V47.4-V47.5; V50.2; V53.6; V55.5; V55.6; V56.0-V56.8; V59.4 (exclude 580; 590; 593.0; 593.7; 598.0; 595; 599.0; 601; 603.1; 604; 607.2; 608.0; 608.4; 614; 616.0-616.4) |
| Neoplasm | 140-239; V10; V16; V58.0-V58.1; V66.1-V66.2; V67.1-V67.2; V71.1; V76 (exclude 228.0-228.1) |
| Central nervous system | 331-359.9 (exclude 335.0; 357.0; 359.0-359.3) |
| Pregnancy/newborn | 630-677; V22-V29; V72.4 (exclude 646.2; 646.6; 648.5; 654.0; 654.6; 654.8; 675) |
| Birth admission | V30-V39 |

Further information on each of the ICD 9 codes is available from this website

www.cms.gov/medicare-coverage-database/.../**icd**-**9**-code-lookup.aspx
