# Supplementary material for: Association of Gestational Age at Birth with Reasons for Subsequent Hospitalisation: 18 Years of Follow-Up in a Western Australian Population Study
Source: PLoS One. 2015 Jun 26;10(6):e0130535. doi: 10.1371/journal.pone.0130535 (PMC4482718; doi:10.1371/journal.pone.0130535)
Supplement: S2 Appendix — (DOCX) [file pone.0130535.s002.docx]

Table 4: Risk of admission to hospital from birth to 28d by principal diagnosis and gestational age

| Diagnosis | Gestational age (weeks) | | | | | | | | | |
| --- | --- | --- | --- | --- | --- | --- | --- | --- | --- | --- |
|  | ≥39 (n=507,677) | | 37-38 (n=206,978) | | 34-36 (n=37,977) | | 32-33 (n=5576) | | <32 (n=6855) | |
|  | n admitted | RR | n admitted | RR (95%CI) | n admitted | RR (95%CI) | n admitted | RR (95%CI) | n admitted | RR (95%CI) |
| All categories | 57239 | 1 | 35917 | 1.48 (1.46-1.49) | 19272 | 4.24 (4.19-4.29) | 4993 | 7.36 (7.27-7.44) | 6167 | 7.35 (7.27-7.44) |
| Infection | 7607 | 1 | 3411 | 1.07 (1.03-1.11) | 656 | 1.12 (1.04-1.22) | 57 | 0.67 (0.52-0.87) | 12 | 0.12 (0.07-0.2) |
| Respiratory | 431 | 1 | 223 | 1.35 (1.15-1.59) | 70 | 2.25 (1.75-2.89) | 7 | 1.5 (0.71-3.17) | 3 | 0.52 (0.17-1.63) |
| Gastrointestinal | 1563 | 1 | 837 | 1.34 (1.23-1.45) | 212 | 1.81 (1.57-2.09) | 22 | 1.28 (0.84-1.94) | 5 | 0.24 (0.1-0.57) |
| Oral | 21 | 1 | 11 | 1.37 (0.66-2.85) | 3 | 1.96 (0.58-6.57) | 0 | na | 0 | na |
| Perinatal | 33880 | 1 | 25549 | 1.74 (1.72-1.77) | 17665 | 6.51 (6.41-6.61) | 4825 | 11.89 (11.72-12.06) | 6071 | 12.07 (11.9-12.24) |
| Congenital | 4967 | 1 | 2568 | 1.3 (1.24-1.37) | 584 | 1.58 (1.45-1.71) | 105 | 1.92 (1.59-2.33) | 74 | 1.12 (0.89-1.4) |
| Social | 2614 | 1 | 1211 | 1.31 (1.22-1.4) | 222 | 1.24 (1.08-1.42) | 32 | 1.16 (0.82-1.64) | 11 | 0.32 (0.18-0.58) |
| Mental | 162 | 1 | 70 | 0.92 (0.69-1.21) | 9 | 0.68 (0.35-1.33) | 0 | na | 1 | 0.43 (0.06-3.04) |
| Injury | 317 | 1 | 154 | 1.19 (0.99-1.45) | 32 | 1.33 (0.93-1.92) | 2 | 0.57 (0.14-2.28) | 1 | 0.23 (0.03-1.63) |
| Renal | 3727 | 1 | 1383 | 0.91 (0.85-0.97) | 151 | 0.51 (0.44-0.61) | 8 | 0.18 (0.09-0.37) | 0 | na |
| Neoplasm | 48 | 1 | 18 | 0.93 (0.54-1.6) | 6 | 1.67 (0.71-3.9) | 1 | 1.91 (0.26-13.81) | 3 | 4.7 (1.46-15.09) |
| CNS | 71 | 1 | 48 | 1.5 (1.04-2.17) | 10 | 1.77 (0.91-3.43) | 0 | na | 1 | 1.01 (0.14-7.26) |

Sample = cohort at birth

p-value: test of overall association between preterm birth and risk of hospitalisation for each diagnostic category RR adjusted for sex and year of birth

Table 5 : Risk of admission to hospital from 29d-1y by principal diagnosis and gestational age

| Diagnosis | Gestational age (weeks) | | | | | | | | | |
| --- | --- | --- | --- | --- | --- | --- | --- | --- | --- | --- |
|  | ≥39 (n=505,829) | | 37-38 (n=205,871) | | 34-36 (n=37,604) | | 32-33 (n=5427) | | <32 (n=5745) | |
|  | n admitted | RR | n admitted | RR (95%CI) | n admitted | RR (95%CI) | n admitted | RR (95%CI) | n admitted | RR (95%CI) |
| All categories | 76281 | 1 | 38011 | 1.23 (1.22-1.24) | 9794 | 1.71 (1.68-1.75) | 1879 | 2.27 (2.18-2.35) | 2787 | 3.16 (3.07-3.24) |
| Infection | 39011 | 1 | 20069 | 1.28 (1.26-1.3) | 5526 | 1.9 (1.85-1.95) | 1053 | 2.5 (2.37-2.64) | 1706 | 3.82 (3.67-3.98) |
| Respiratory | 6109 | 1 | 3315 | 1.43 (1.37-1.49) | 1113 | 2.52 (2.37-2.69) | 247 | 3.82 (3.37-4.32) | 535 | 7.91 (7.27-8.6) |
| Gastrointestinal | 10742 | 1 | 5938 | 1.44 (1.4-1.49) | 1981 | 2.54 (2.42-2.66) | 488 | 4.26 (3.91-4.64) | 745 | 6.23 (5.82-6.68) |
| Oral | 259 | 1 | 113 | 1.16 (0.93-1.45) | 26 | 1.41 (0.94-2.11) | 0 | n/a | 5 | 1.76 (0.73-4.27) |
| Perinatal | 304 | 1 | 223 | 1.65 (1.39-1.96) | 119 | 5 (4.05-6.18) | 51 | 15.17 (11.29-20.38) | 156 | 43.03 (35.54-52.11) |
| Congenital | 7023 | 1 | 3634 | 1.22 (1.18-1.27) | 918 | 1.69 (1.58-1.81) | 177 | 2.28 (1.97-2.64) | 201 | 2.43 (2.12-2.79) |
| Social | 4712 | 1 | 1721 | 1.2 (1.14-1.27) | 516 | 1.74 (1.59-1.9) | 88 | 1.9 (1.55-2.34) | 118 | 2.5 (2.09-2.99) |
| Mental | 2946 | 1 | 1775 | 1.2 (1.13-1.27) | 310 | 1.24 (1.1-1.39) | 42 | 1.21 (0.89-1.63) | 44 | 1.13 (0.84-1.52) |
| Injury | 5107 | 1 | 2226 | 1.09 (1.04-1.15) | 505 | 1.34 (1.22-1.46) | 95 | 1.74 (1.42-2.12) | 151 | 2.62 (2.23-3.07) |
| Renal | 3489 | 1 | 1745 | 1.15 (1.08-1.21) | 476 | 1.68 (1.53-1.85) | 74 | 1.83 (1.45-2.29) | 55 | 1.27 (0.98-1.66) |
| Neoplasm | 538 | 1 | 246 | 1.09 (0.93-1.26) | 38 | 0.93 (0.67-1.29) | 7 | 1.2 (0.57-2.53) | 4 | 0.64 (0.24-1.72) |
| CNS | 416 | 1 | 231 | 1.37 (1.16-1.61) | 84 | 2.7 (2.14-3.41) | 12 | 2.68 (1.51-4.75) | 38 | 8.05 (5.78-11.2) |

Sample = cohort alive at 29d

p-value: test of overall association between preterm birth and risk of hospitalisation for each diagnostic category

RR adjusted for sex and year of birth

.

Table 6 : Risk of admission to hospital from 1-5y by principal diagnosis and gestational age

| Diagnosis | Gestational age (weeks) | | | | | | | | | |
| --- | --- | --- | --- | --- | --- | --- | --- | --- | --- | --- |
|  | ≥39 (n=487,901) | | 37-38 (n=196,468) | | 34-36 (n=35,874) | | 32-33 (n=5194) | | <32 (n=5366) | |
|  | n admitted | RR | n admitted | RR (95%CI) | n admitted | RR (95%CI) | n admitted | RR (95%CI) | n admitted | RR (95%CI) |
| All categories | 152429 | 1 | 66986 | 1.11 (1.1-1.11) | 14161 | 1.26 (1.24-1.28) | 2309 | 1.42 (1.37-1.46) | 2933 | 1.73 (1.69-1.77) |
| Infection | 79014 | 1 | 36789 | 1.17 (1.16-1.18) | 8193 | 1.41 (1.38-1.44) | 1316 | 1.56 (1.49-1.64) | 1935 | 2.22 (2.14-2.3) |
| Respiratory | 19065 | 1 | 8679 | 1.18 (1.15-1.21) | 2142 | 1.55 (1.48-1.62) | 450 | 2.23 (2.04-2.44) | 807 | 3.88 (3.64-4.14) |
| Gastrointestinal | 13499 | 1 | 6072 | 1.22 (1.18-1.25) | 1652 | 1.73 (1.65-1.82) | 273 | 1.94 (1.73-2.18) | 389 | 2.73 (2.48-3) |
| Oral | 14133 | 1 | 5935 | 1.03 (1-1.06) | 1253 | 1.19 (1.12-1.26) | 171 | 1.13 (0.98-1.31) | 185 | 1.19 (1.03-1.37) |
| Congenital | 8506 | 1 | 4016 | 1.18 (1.14-1.22) | 944 | 1.49 (1.39-1.59) | 199 | 2.16 (1.89-2.48) | 300 | 3.17 (2.84-3.55) |
| Social | 1685 | 1 | 771 | 1.31 (1.21-1.43) | 254 | 2.22 (1.95-2.53) | 57 | 3.34 (2.57-4.34) | 90 | 5.18 (4.2-6.38) |
| Mental | 540 | 1 | 254 | 1.23 (1.06-1.43) | 55 | 1.41 (1.06-1.85) | 13 | 2.28 (1.32-3.95) | 19 | 3.28 (2.08-5.18) |
| Injury | 32202 | 1 | 13444 | 1.07 (1.05-1.09) | 2706 | 1.15 (1.11-1.2) | 429 | 1.26 (1.15-1.38) | 494 | 1.42 (1.3-1.54) |
| Renal | 8689 | 1 | 3862 | 1.1 (1.06-1.14) | 861 | 1.29 (1.2-1.38) | 156 | 1.6 (1.37-1.87) | 184 | 1.85 (1.61-2.13) |
| Neoplasm | 1727 | 1 | 748 | 1.09 (1-1.19) | 126 | 1 (0.83-1.2) | 22 | 1.21 (0.79-1.84) | 27 | 1.44 (0.99-2.1) |
| CNS | 1101 | 1 | 552 | 1.27 (1.15-1.41) | 159 | 1.98 (1.67-2.33) | 40 | 3.43 (2.51-4.7) | 100 | 8.38 (6.84-10.26) |

Sample = cohort alive at 1y

p-value: test of overall association between preterm birth and risk of hospitalisation for each diagnostic category

RR adjusted for sex and year of birth

Table 7: Risk of admission to hospital from 5-12y by principal diagnosis and gestational age

| Diagnosis | Gestational age (weeks) | | | | | | | | | |
| --- | --- | --- | --- | --- | --- | --- | --- | --- | --- | --- |
|  | ≥39 (n=417,216) | | 37-38 (n=158,523) | | 34-36 (n=29,649) | | 32-33 (n=4302) | | <32 (n=4392) | |
|  | n admitted | RR | n admitted | RR (95%CI) | n admitted | RR (95%CI) | n admitted | RR (95%CI) | n admitted | RR (95%CI) |
| All categories | 117232 | 1 | 46407 | 1.08 (1.07-1.09) | 9963 | 1.21 (1.19-1.23) | 1519 | 1.25 (1.2-1.3) | 1860 | 1.52 (1.47-1.57) |
| Infection | 49379 | 1 | 19770 | 1.11 (1.09-1.13) | 4536 | 1.32 (1.29-1.36) | 733 | 1.45 (1.36-1.55) | 916 | 1.8 (1.7-1.91) |
| Respiratory | 10210 | 1 | 4055 | 1.14 (1.1-1.18) | 890 | 1.27 (1.19-1.36) | 136 | 1.31 (1.11-1.54) | 238 | 2.26 (2-2.56) |
| Gastrointestinal | 11048 | 1 | 4402 | 1.16 (1.12-1.2) | 1004 | 1.34 (1.26-1.43) | 157 | 1.41 (1.21-1.65) | 200 | 1.79 (1.56-2.05) |
| Oral | 14094 | 1 | 6368 | 1.13 (1.1-1.16) | 1237 | 1.2 (1.13-1.27) | 154 | 1.04 (0.89-1.22) | 220 | 1.45 (1.27-1.65) |
| Congenital | 5226 | 1 | 2194 | 1.16 (1.1-1.22) | 509 | 1.39 (1.27-1.52) | 96 | 1.78 (1.46-2.17) | 146 | 2.69 (2.29-3.16) |
| Social | 660 | 1 | 286 | 1.16 (1.01-1.33) | 76 | 1.62 (1.28-2.06) | 20 | 2.96 (1.9-4.62) | 22 | 3.17 (2.08-4.84) |
| Mental | 973 | 1 | 383 | 1.11 (0.99-1.25) | 92 | 1.36 (1.1-1.68) | 12 | 1.19 (0.68-2.1) | 18 | 1.79 (1.12-2.85) |
| Injury | 29949 | 1 | 11416 | 1.06 (1.04-1.08) | 2511 | 1.2 (1.16-1.25) | 354 | 1.15 (1.04-1.27) | 389 | 1.26 (1.14-1.38) |
| Renal | 6993 | 1 | 2853 | 1.09 (1.04-1.14) | 647 | 1.25 (1.16-1.35) | 97 | 1.28 (1.05-1.56) | 120 | 1.57 (1.32-1.87) |
| Neoplasm | 2293 | 1 | 944 | 1.14 (1.05-1.23) | 159 | 1 (0.85-1.18) | 37 | 1.59 (1.15-2.2) | 23 | 0.98 (0.65-1.48) |
| CNS | 1312 | 1 | 557 | 1.18 (1.07-1.3) | 151 | 1.66 (1.41-1.97) | 31 | 2.32 (1.63-3.31) | 86 | 6.4 (5.16-7.94) |

Sample = cohort alive at 5y

p-value: test of overall association between preterm birth and risk of hospitalisation for each diagnostic category

RR adjusted for sex and year of birth

Table 8: Risk of admission to hospital from 12-18y by principal diagnosis and gestational age

| Diagnosis | Gestational age (weeks) | | | | | | | | | |
| --- | --- | --- | --- | --- | --- | --- | --- | --- | --- | --- |
|  | ≥39 (n=311,526) | | 37-38 (n=105,057) | | 34-36 (n=20,801) | | 32-33 (n=3110) | | <32 (n=3099) | |
|  | n admitted | RR | n admitted | RR (95%CI) | n admitted | RR (95%CI) | n admitted | RR (95%CI) | n admitted | RR (95%CI) |
| All categories | 88517 | 1 | 29404 | 1.03 (1.02-1.04) | 6082 | 1.06 (1.03-1.08) | 919 | 1.05 (1-1.11) | 979 | 1.14 (1.08-1.2) |
| Infection | 19542 | 1 | 6639 | 1.06 (1.03-1.09) | 1489 | 1.18 (1.12-1.24) | 224 | 1.17 (1.03-1.33) | 254 | 1.35 (1.2-1.52) |
| Respiratory | 3647 | 1 | 1267 | 1.11 (1.04-1.18) | 293 | 1.24 (1.11-1.4) | 56 | 1.56 (1.2-2.03) | 64 | 1.81 (1.42-2.31) |
| Gastrointestinal | 7927 | 1 | 2695 | 1.07 (1.03-1.12) | 580 | 1.15 (1.06-1.25) | 104 | 1.35 (1.12-1.64) | 101 | 1.33 (1.1-1.62) |
| Oral | 22737 | 1 | 7494 | 1.03 (1.01-1.06) | 1350 | 0.93 (0.88-0.98) | 179 | 0.81 (0.7-0.94) | 211 | 0.97 (0.86-1.11) |
| Congenital | 2535 | 1 | 847 | 1.04 (0.96-1.12) | 178 | 1.08 (0.93-1.26) | 34 | 1.36 (0.97-1.9) | 47 | 1.91 (1.44-2.54) |
| Social | 340 | 1 | 132 | 1.13 (0.92-1.38) | 34 | 1.47 (1.03-2.09) | 7 | 2.03 (0.96-4.29) | 12 | 3.54 (1.99-6.28) |
| Mental | 4167 | 1 | 1424 | 1.07 (1-1.13) | 344 | 1.29 (1.15-1.43) | 40 | 0.98 (0.72-1.34) | 59 | 1.48 (1.15-1.91) |
| Injury | 24476 | 1 | 8036 | 1.01 (0.98-1.03) | 1698 | 1.04 (0.99-1.09) | 285 | 1.15 (1.03-1.28) | 263 | 1.07 (0.96-1.2) |
| Renal | 4130 | 1 | 1535 | 1.16 (1.09-1.22) | 321 | 1.19 (1.06-1.33) | 47 | 1.15 (0.86-1.53) | 55 | 1.36 (1.05-1.78) |
| Neoplasm | 2698 | 1 | 834 | 0.96 (0.88-1.03) | 159 | 0.91 (0.78-1.07) | 21 | 0.8 (0.52-1.22) | 22 | 0.85 (0.56-1.29) |
| CNS | 1068 | 1 | 443 | 1.3 (1.16-1.45) | 123 | 1.79 (1.49-2.16) | 21 | 2.01 (1.31-3.1) | 54 | 5.26 (4.01-6.9) |
| Pregnancy | 6238 | 1 | 1986 | 1.07 (1.02-1.12) | 484 | 1.33 (1.21-1.45) | 63 | 1.12 (0.88-1.42) | 51 | 0.93 (0.71-1.22) |

Sample = cohort alive at 12y

p-value: test of overall association between preterm birth and risk of hospitalisation for each diagnostic category

RR adjusted for sex and year of birth
