# Supplementary material for: Association of Gestational Age at Birth with Reasons for Subsequent Hospitalisation: 18 Years of Follow-Up in a Western Australian Population Study
Source: PLoS One. 2015 Jun 26;10(6):e0130535. doi: 10.1371/journal.pone.0130535 (PMC4482718; doi:10.1371/journal.pone.0130535)
Supplement: S3 Appendix — (DOCX) [file pone.0130535.s003.docx]

Figure 4: Most common diagnoses associated with non-infectious respiratory admissions 29 days -1 year and 1-5 years

| Figure 4 A: Post-neonatal period: 29 days to 1 year | Figure 4 B: Infancy: 1-5 years |
| --- | --- |
|  |  |

Figure 5 Most common diagnoses associated with gastrointestinal admissions 29 days-1year and 1-5 years

| Figure 5 A: Post-neonatal period: 29d to 1y | Figure 5 B: Infancy: 1-5y |
| --- | --- |
|  |  |

Figure 6: Most common diagnoses associated with CNS admissions 29 days-1year, 1-5 years, 5-12 years and 12-18 years

| Figure 6 A: Post-neonatal: 29 days – 1 year | Figure 6 B: Infancy: 1-5 years |
| --- | --- |
|  |  |
| Figure 6 C: Childhood: 5-12y | Figure 6 D: Adolescence: 12-18y |
|  |  |
