# Supplementary material for: Association of Gestational Age at Birth with Reasons for Subsequent Hospitalisation: 18 Years of Follow-Up in a Western Australian Population Study
Source: PLoS One. 2015 Jun 26;10(6):e0130535. doi: 10.1371/journal.pone.0130535 (PMC4482718; doi:10.1371/journal.pone.0130535)
Supplement: S1 Table — (DOCX) [file pone.0130535.s004.docx]

**S1 Table: Hospital admissions per 100 live, singleton births 1980-2010 over different childhood ages according to gestational age**

|  | Age period of follow-up | | | | | | | | | | | |
| --- | --- | --- | --- | --- | --- | --- | --- | --- | --- | --- | --- | --- |
|  | 0-28 days | | 29days-1year | | 1-5 years | | 5-12 years | | 12-18 years | | 0-18 years | |
| Gestational age | n* | % admitted | n* | % admitted | n* | % admitted | n* | % admitted | n* | % admitted | n* | % admitted |
| ≥39 wks | 507677 | 11.3 | 505829 | 15.1 | 487901 | 31.2 | 417216 | 28.1 | 311526 | 28.4 | 507677 | 59.5 |
| 37-38wks | 206978 | 17.4 | 205871 | 18.5 | 196468 | 34.1 | 158523 | 29.3 | 105057 | 28 | 206978 | 62.4 |
| 34-36wks | 37977 | 50.7 | 37604 | 26 | 35874 | 39.5 | 29649 | 33.6 | 20801 | 29.2 | 37977 | 80.8 |
| 32-33wks | 5576 | 89.5 | 5427 | 34.6 | 5194 | 44.5 | 4302 | 35.3 | 3110 | 29.5 | 5576 | 96.5 |
| <32 wks | 6855 | 90 | 5745 | 48.5 | 5366 | 54.7 | 4392 | 42.3 | 3099 | 31.6 | 6855 | 93.6 |
| Total | 765063 | 16.2 | 760476 | 16.9 | 730803 | 32.7 | 614082 | 28.8 | 443593 | 28.4 | 765063 | 61.9 |

*cohort alive at start of each period of follow up
