# Supplementary material for: Association of Gestational Age at Birth with Reasons for Subsequent Hospitalisation: 18 Years of Follow-Up in a Western Australian Population Study
Source: PLoS One. 2015 Jun 26;10(6):e0130535. doi: 10.1371/journal.pone.0130535 (PMC4482718; doi:10.1371/journal.pone.0130535)
Supplement: S2 Table — (DOCX) [file pone.0130535.s005.docx]

**S2 Table: Risk and causes for admission to hospital from birth to 28 days and 29 days-1 year by principal diagnosis and gestational age**

| Diagnosis | Gestational age (weeks) | | | | | | | | | |
| --- | --- | --- | --- | --- | --- | --- | --- | --- | --- | --- |
| **0-28 days** | **≥39 (n=507,677)** | | **37-38 (n=206,978)** | | **34-36 (n=37,977)** | | **32-33 (n=5576)** | | **<32 (n=6855)** | |
|  | n admitted | RR | n admitted | RR (95%CI) | n admitted | RR (95%CI) | n admitted | RR (95%CI) | n admitted | RR (95%CI) |
| All categories | 57239 | 1 | 35917 | 1.48 (1.46-1.49) | 19272 | 4.24 (4.19-4.29) | 4993 | 7.36 (7.27-7.44) | 6167 | 7.35 (7.27-7.44) |
| Perinatal | 33880 | 1 | 25549 | 1.74 (1.72-1.77) | 17665 | 6.51 (6.41-6.61) | 4825 | 11.89 (11.72-12.06) | 6071 | 12.07 (11.9-12.24) |
| Infection | 7607 | 1 | 3411 | 1.07 (1.03-1.11) | 656 | 1.12 (1.04-1.22) | 57 | 0.67 (0.52-0.87) | 12 | 0.12 (0.07-0.2) |
| Congenital | 4967 | 1 | 2568 | 1.3 (1.24-1.37) | 584 | 1.58 (1.45-1.71) | 105 | 1.92 (1.59-2.33) | 74 | 1.12 (0.89-1.4) |
| **29 days- 1 year** | **n=505,829** |  | **n=205,871** |  | **n=37,604** |  | **n=5,427** |  | **n=5,745** |  |
| All categories | 76281 | 1 | 38011 | 1.23 (1.22-1.24) | 9794 | 1.71 (1.68-1.75) | 1879 | 2.27 (2.18-2.35) | 2787 | 3.16 (3.07-3.24) |
| Infection | 39011 | 1 | 20069 | 1.28 (1.26-1.3) | 5526 | 1.9 (1.85-1.95) | 1053 | 2.5 (2.37-2.64) | 1706 | 3.82 (3.67-3.98) |
| Gastrointestinal | 10742 | 1 | 5938 | 1.44 (1.4-1.49) | 1981 | 2.54 (2.42-2.66) | 488 | 4.26 (3.91-4.64) | 745 | 6.23 (5.82-6.68) |
| Congenital | 7023 | 1 | 3634 | 1.22 (1.18-1.27) | 918 | 1.69 (1.58-1.81) | 177 | 2.28 (1.97-2.64) | 201 | 2.43 (2.12-2.79) |

Sample = cohort at birth

RR, risk ratio

RR adjusted for sex and year of birth
