# Supplementary material for: Association of Gestational Age at Birth with Reasons for Subsequent Hospitalisation: 18 Years of Follow-Up in a Western Australian Population Study
Source: PLoS One. 2015 Jun 26;10(6):e0130535. doi: 10.1371/journal.pone.0130535 (PMC4482718; doi:10.1371/journal.pone.0130535)
Supplement: S3 Table — (DOCX) [file pone.0130535.s006.docx]

**S3 Table: Risk and causes for admission to hospital from 1-18 years by principal diagnosis and gestational age**

| Diagnosis | Gestational age (weeks) | | | | | | | | | |
| --- | --- | --- | --- | --- | --- | --- | --- | --- | --- | --- |
| **1-5 years** | **≥39 (n=487,901)** | | **37-38 (n=196,468)** | | **34-36 (n=35,874)** | | **32-33 (n=5194)** | | **<32 (n=5366)** | |
|  | n admitted | RR | n admitted | RR (95%CI) | n admitted | RR (95%CI) | n admitted | RR (95%CI) | n admitted | RR (95%CI) |
| All categories | 152429 | 1 | 66986 | 1.11 (1.1-1.11) | 14161 | 1.26 (1.24-1.28) | 2309 | 1.42 (1.37-1.46) | 2933 | 1.73 (1.69-1.77) |
| Infection | 79014 | 1 | 36789 | 1.17 (1.16-1.18) | 8193 | 1.41 (1.38-1.44) | 1316 | 1.56 (1.49-1.64) | 1935 | 2.22 (2.14-2.3) |
| Injury | 32202 | 1 | 13444 | 1.07 (1.05-1.09) | 2706 | 1.15 (1.11-1.2) | 429 | 1.26 (1.15-1.38) | 494 | 1.42 (1.3-1.54) |
| Respiratory | 19065 | 1 | 8679 | 1.18 (1.15-1.21) | 2142 | 1.55 (1.48-1.62) | 450 | 2.23 (2.04-2.44) | 807 | 3.88 (3.64-4.14) |
| **Age 5-12 years** | **n=417,219** | **1** | **n=158,523** |  | **n=29,649** |  | **n=4,302** |  | **n=4,392** |  |
| All categories | 117232 | 1 | 46407 | 1.08 (1.07-1.09) | 9963 | 1.21 (1.19-1.23) | 1519 | 1.25 (1.2-1.3) | 1860 | 1.52 (1.47-1.57) |
| Infection | 49379 | 1 | 19770 | 1.11 (1.09-1.13) | 4536 | 1.32 (1.29-1.36) | 733 | 1.45 (1.36-1.55) | 916 | 1.8 (1.7-1.91) |
| Injury | 29949 | 1 | 11416 | 1.06 (1.04-1.08) | 2511 | 1.2 (1.16-1.25) | 354 | 1.15 (1.04-1.27) | 389 | 1.26 (1.14-1.38) |
| Gastrointestinal | 11048 | 1 | 4402 | 1.16 (1.12-1.2) | 1004 | 1.34 (1.26-1.43) | 157 | 1.41 (1.21-1.65) | 200 | 1.79 (1.56-2.05) |
| **Age 12-18 years** | **N=311,526** |  | **N=105,057** |  | **N=20,801** |  | **N=3,110** |  | **N=3,099** |  |
| All categories | 88517 | 1 | 29404 | 1.03 (1.02-1.04) | 6082 | 1.06 (1.03-1.08) | 919 | 1.05 (1-1.11) | 979 | 1.14 (1.08-1.2) |
| Injury | 24476 | 1 | 8036 | 1.01 (0.98-1.03) | 1698 | 1.04 (0.99-1.09) | 285 | 1.15 (1.03-1.28) | 263 | 1.07 (0.96-1.2) |
| Oral | 22737 | 1 | 7494 | 1.03 (1.01-1.06) | 1350 | 0.93 (0.88-0.98) | 179 | 0.81 (0.7-0.94) | 211 | 0.97 (0.86-1.11) |
| Infection | 19542 | 1 | 6639 | 1.06 (1.03-1.09) | 1489 | 1.18 (1.12-1.24) | 224 | 1.17 (1.03-1.33) | 254 | 1.35 (1.2-1.52) |

Sample = cohort alive at 1y

RR, risk ratio

RR adjusted for sex and year of birth
